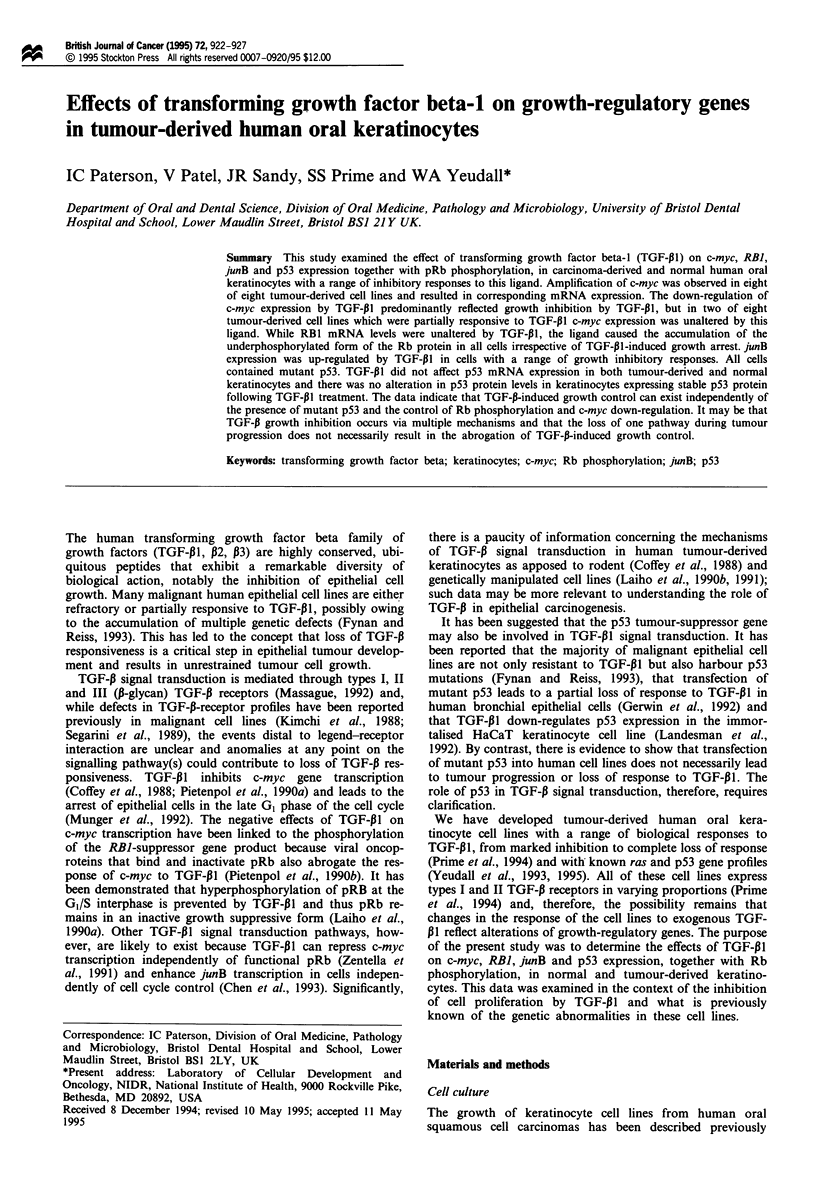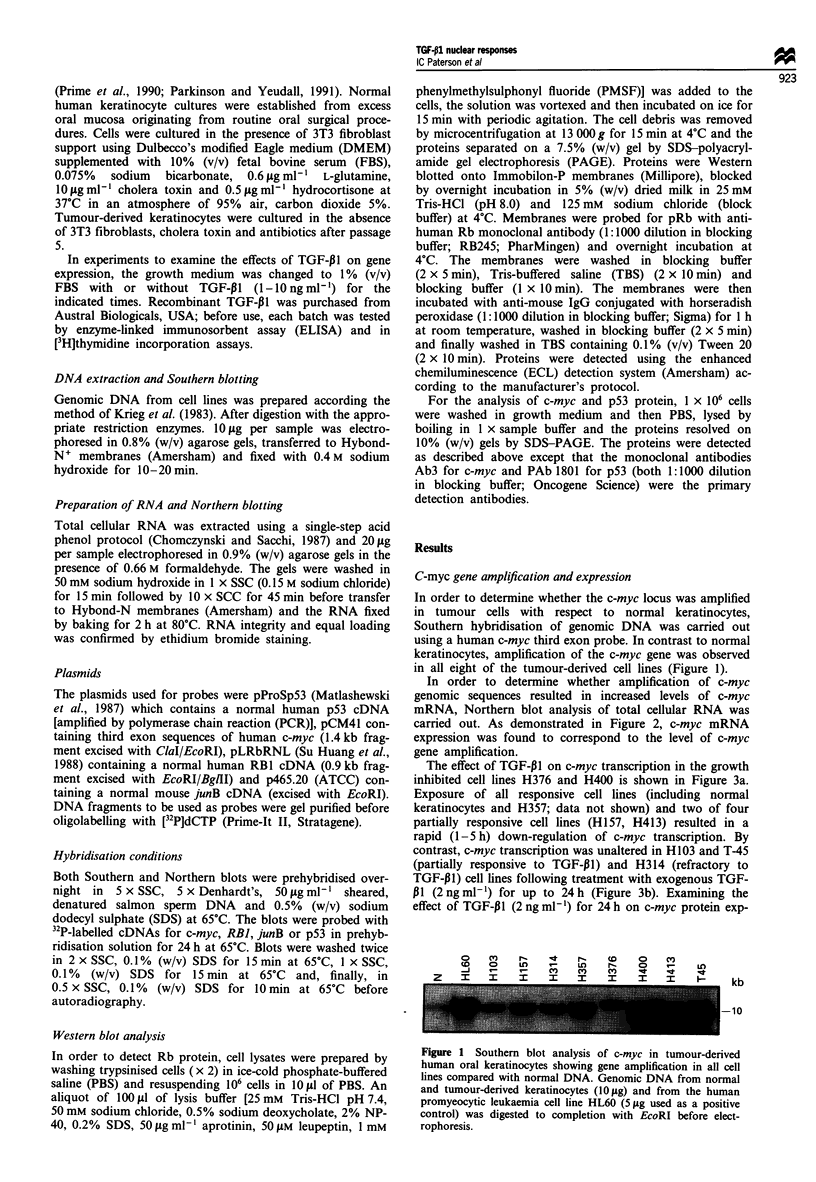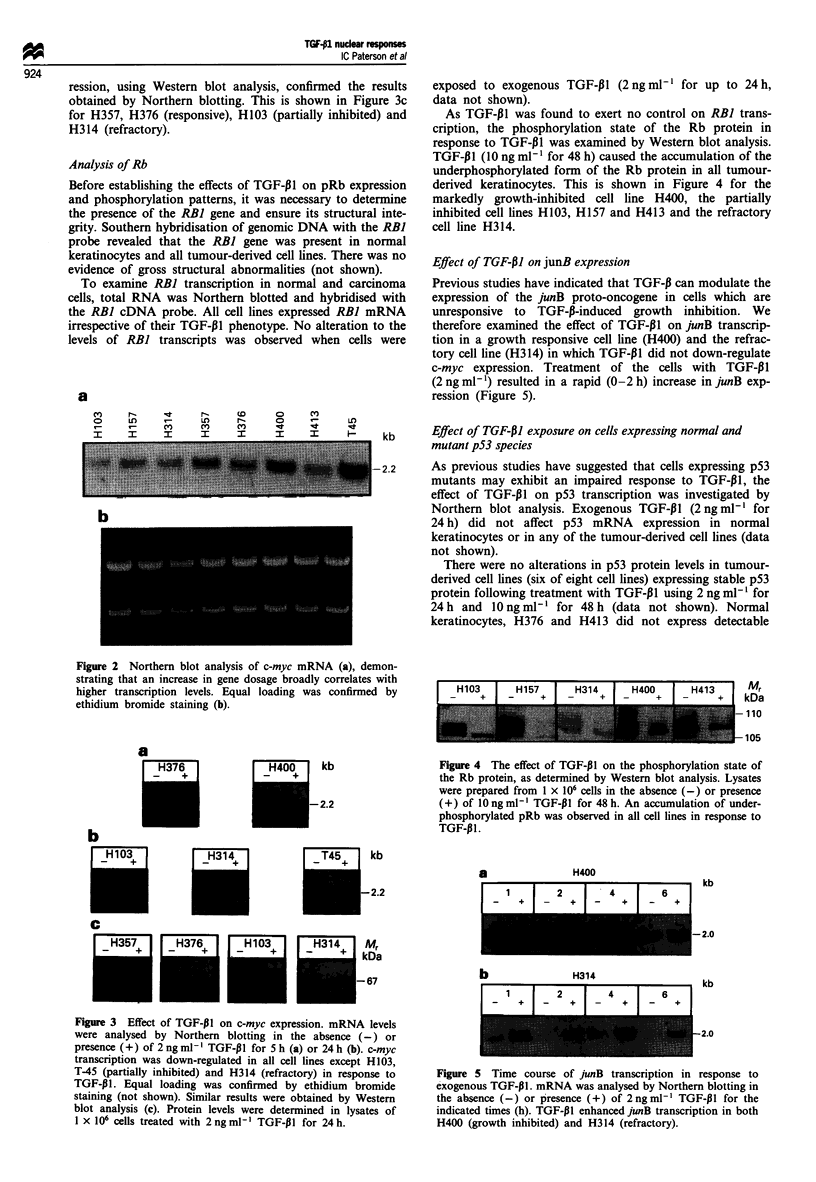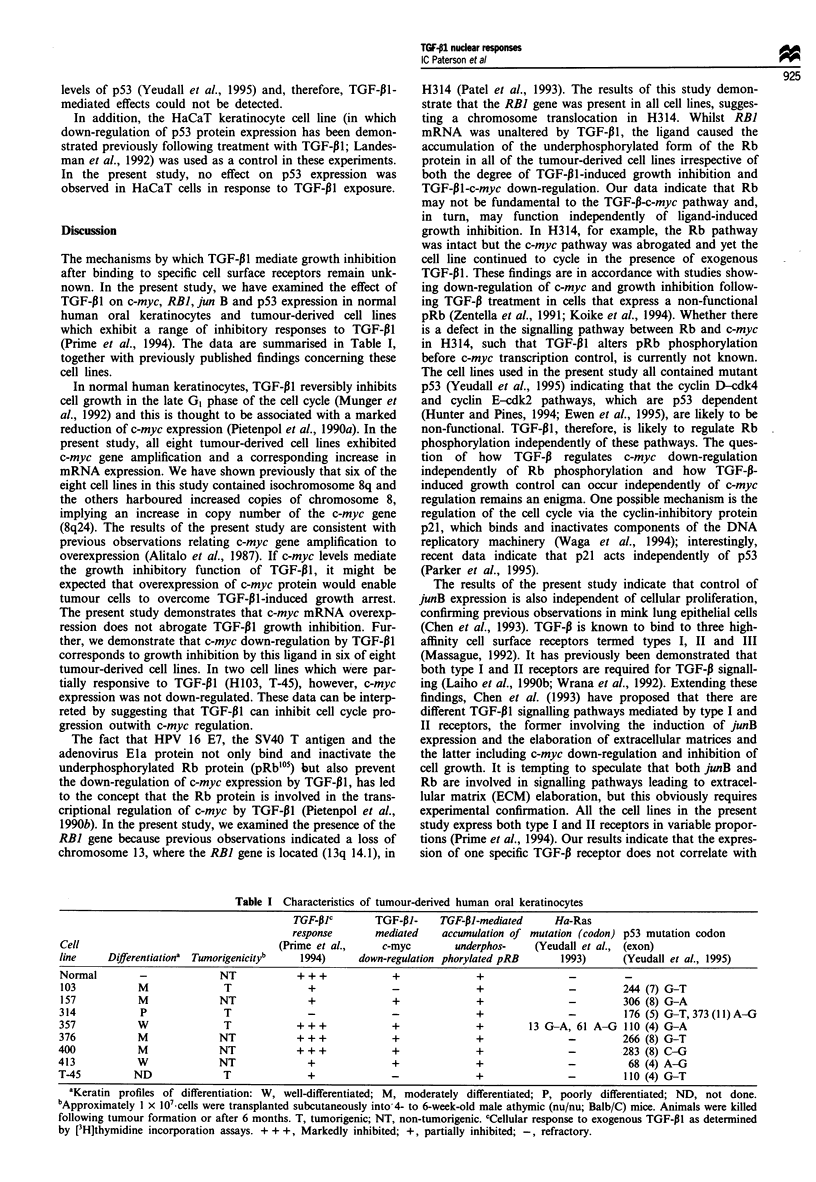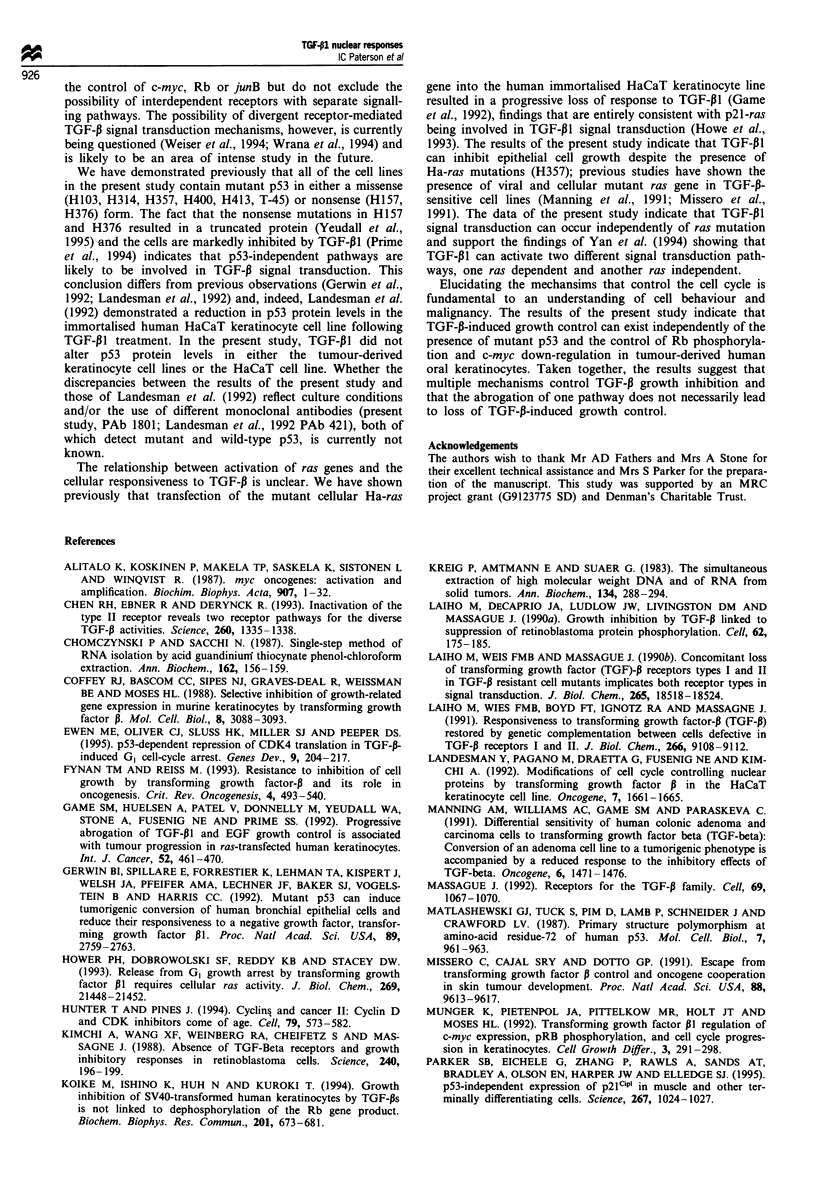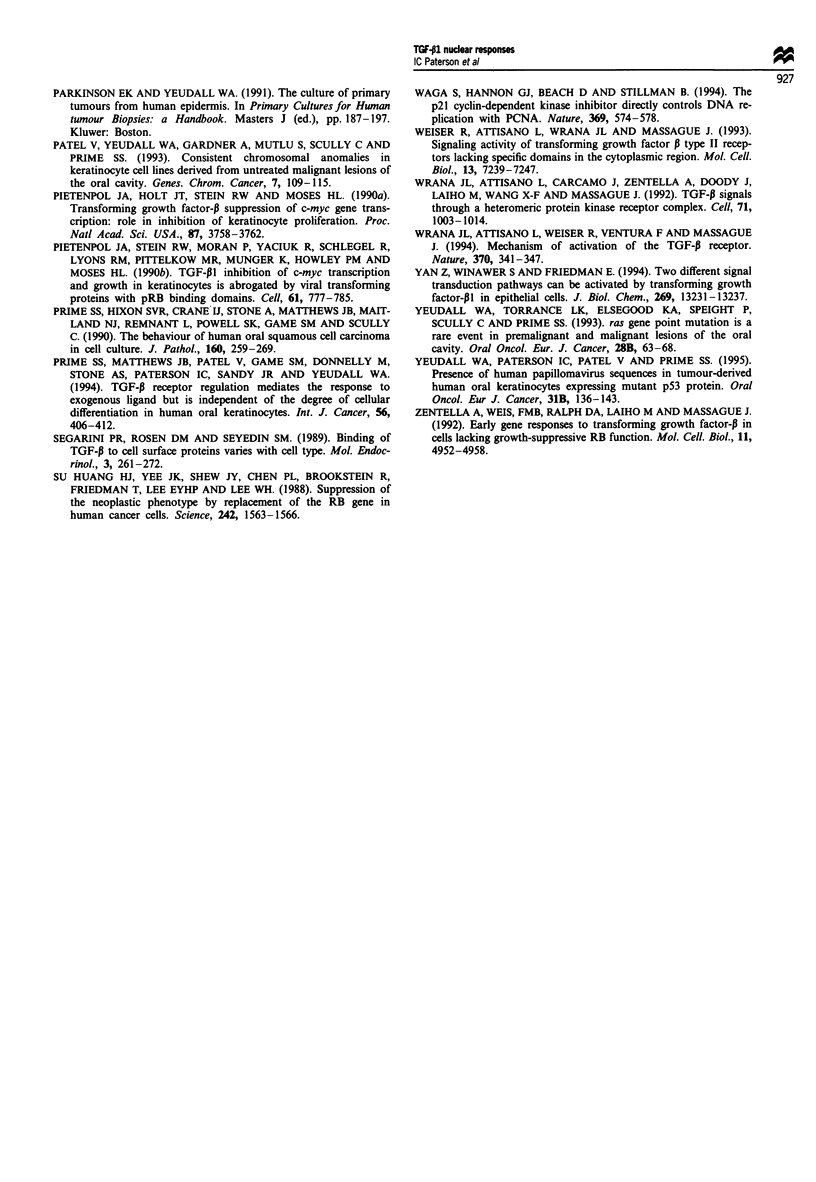# Effects of transforming growth factor beta-1 on growth-regulatory genes in tumour-derived human oral keratinocytes.

**DOI:** 10.1038/bjc.1995.434

**Published:** 1995-10

**Authors:** I. C. Paterson, V. Patel, J. R. Sandy, S. S. Prime, W. A. Yeudall

**Affiliations:** Department of Oral and Dental Science, University of Bristol Dental Hospital and School, UK.

## Abstract

**Images:**


British Journal of Cancer (1995) 72, 922-927
© 1995 Stockton Press All rights reserved 0007-0920/95 $12.00

### Effects of transforming growth factor beta-1 on growth-regulatory genes in tumour-derived human oral keratinocytes

IC Paterson, V Patel, JR Sandy, SS Prime and WA Yeudall*

*Department of Oral and Dental Science, Division of Oral Medicine, Pathology and Microbiology, University of Bristol Dental Hospital and School, Lower Maudlin Street, Bristol BS1 2lY UK.*

**Summary** This study examined the effect of transforming growth factor beta-1 (TGF-β1) on c-*myc*, *RB1*, *jun*B and p53 expression together with pRb phosphorylation, in carcinoma-derived and normal human oral keratinocytes with a range of inhibitory responses to this ligand. Amplification of c-*myc* was observed in eight of eight tumour-derived cell lines and resulted in corresponding mRNA expression. The down-regulation of c-*myc* expression by TGF-β1 predominantly reflected growth inhibition by TGF-β1, but in two of eight tumour-derived cell lines which were partially responsive to TGF-β1 c-*myc* expression was unaltered by this ligand. While RB1 mRNA levels were unaltered by TGF-β1, the ligand caused the accumulation of the underphosphorylated form of the Rb protein in all cells irrespective of TGF-β1-induced growth arrest. *jun*B expression was up-regulated by TGF-β1 in cells with a range of growth inhibitory responses. All cells contained mutant p53. TGF-β1 did not affect p53 mRNA expression in both tumour-derived and normal keratinocytes and there was no alteration in p53 protein levels in keratinocytes expressing stable p53 protein following TGF-β1 treatment. The data indicate that TGF-β-induced growth control can exist independently of the presence of mutant p53 and the control of Rb phosphorylation and c-*myc* down-regulation. It may be that TGF-β growth inhibition occurs via multiple mechanisms and that the loss of one pathway during tumour progression does not necessarily result in the abrogation of TGF-β-induced growth control.

**Keywords:** transforming growth factor beta; keratinocytes; c-*myc*; Rb phosphorylation; *jun*B; p53

The human transforming growth factor beta family of growth factors (TGF-β1, β2, β3) are highly conserved, ubiquitous peptides that exhibit a remarkable diversity of biological action, notably the inhibition of epithelial cell growth. Many malignant human epithelial cell lines are either refractory or partially responsive to TGF-β1, possibly owing to the accumulation of multiple genetic defects (Fynan and Reiss, 1993). This has led to the concept that loss of TGF-β responsiveness is a critical step in epithelial tumour development and results in unrestrained tumour cell growth.

TGF-β signal transduction is mediated through types I, II and III (β-glycan) TGF-β receptors (Massague, 1992) and, while defects in TGF-β-receptor profiles have been reported previously in malignant cell lines (Kimchi *et al.*, 1988; Segarini *et al.*, 1989), the events distal to legend–receptor interaction are unclear and anomalies at any point on the signalling pathway(s) could contribute to loss of TGF-β responsiveness. TGF-β1 inhibits c-*myc* gene transcription (Coffey *et al.*, 1988; Pietenpol *et al.*, 1990*a*) and leads to the arrest of epithelial cells in the late $G_1$ phase of the cell cycle (Munger *et al.*, 1992). The negative effects of TGF-β1 on c-*myc* transcription have been linked to the phosphorylation of the *RB1*-suppressor gene product because viral oncoproteins that bind and inactivate pRb also abrogate the response of c-*myc* to TGF-β1 (Pietenpol *et al.*, 1990*b*). It has been demonstrated that hyperphosphorylation of pRB at the $G_1$/S interphase is prevented by TGF-β1 and thus pRb remains in an inactive growth suppressive form (Laiho *et al.*, 1990*a*). Other TGF-β1 signal transduction pathways, however, are likely to exist because TGF-β1 can repress c-*myc* transcription independently of functional pRb (Zentella *et al.*, 1991) and enhance *jun*B transcription in cells independently of cell cycle control (Chen *et al.*, 1993). Significantly, there is a paucity of information concerning the mechanisms of TGF-β signal transduction in human tumour-derived keratinocytes as apposed to rodent (Coffey *et al.*, 1988) and genetically manipulated cell lines (Laiho *et al.*, 1990*b*, 1991); such data may be more relevant to understanding the role of TGF-β in epithelial carcinogenesis.

It has been suggested that the p53 tumour-suppressor gene may also be involved in TGF-β1 signal transduction. It has been reported that the majority of malignant epithelial cell lines are not only resistant to TGF-β1 but also harbour p53 mutations (Fynan and Reiss, 1993), that transfection of mutant p53 leads to a partial loss of response to TGF-β1 in human bronchial epithelial cells (Gerwin *et al.*, 1992) and that TGF-β1 down-regulates p53 expression in the immortalised HaCaT keratinocyte cell line (Landesman *et al.*, 1992). By contrast, there is evidence to show that transfection of mutant p53 into human cell lines does not necessarily lead to tumour progression or loss of response to TGF-β1. The role of p53 in TGF-β signal transduction, therefore, requires clarification.

We have developed tumour-derived human oral keratinocyte cell lines with a range of biological responses to TGF-β1, from marked inhibition to complete loss of response (Prime *et al.*, 1994) and with known *ras* and p53 gene profiles (Yeudall *et al.*, 1993, 1995). All of these cell lines express types I and II TGF-β receptors in varying proportions (Prime *et al.*, 1994) and, therefore, the possibility remains that changes in the response of the cell lines to exogenous TGF-β1 reflect alterations of growth-regulatory genes. The purpose of the present study was to determine the effects of TGF-β1 on c-*myc*, *RB1*, *jun*B and p53 expression, together with Rb phosphorylation, in normal and tumour-derived keratinocytes. This data was examined in the context of the inhibition of cell proliferation by TGF-β1 and what is previously known of the genetic abnormalities in these cell lines.

### Materials and methods

### *Cell culture*

The growth of keratinocyte cell lines from human oral squamous cell carcinomas has been described previously

Correspondence: IC Paterson, Division of Oral Medicine, Pathology and Microbiology, Bristol Dental Hospital and School, Lower Maudlin Street, Bristol BS1 2LY, UK
*Present address: Laboratory of Cellular Development and Oncology, NIDR, National Institute of Health, 9000 Rockville Pike, Bethesda, MD 20892, USA
Received 8 December 1994; revised 10 May 1995; accepted 11 May 1995

(Prime et al., 1990; Parkinson and Yeudall, 1991). Normal human keratinocyte cultures were established from excess oral mucosa originating from routine oral surgical procedures. Cells were cultured in the presence of 3T3 fibroblast support using Dulbecco's modified Eagle medium (DMEM) supplemented with 10% (v/v) fetal bovine serum (FBS), 0.075% sodium bicarbonate, 0.6 μg ml$^{-1}$ L-glutamine, 10 μg ml$^{-1}$ cholera toxin and 0.5 μg ml$^{-1}$ hydrocortisone at 37°C in an atmosphere of 95% air, carbon dioxide 5%. Tumour-derived keratinocytes were cultured in the absence of 3T3 fibroblasts, cholera toxin and antibiotics after passage 5.

In experiments to examine the effects of TGF-β1 on gene expression, the growth medium was changed to 1% (v/v) FBS with or without TGF-β1 (1–10 ng ml$^{-1}$) for the indicated times. Recombinant TGF-β1 was purchased from Austral Biologicals, USA; before use, each batch was tested by enzyme-linked immunosorbent assay (ELISA) and in [$^3$H]thymidine incorporation assays.

### *DNA extraction and Southern blotting*

Genomic DNA from cell lines was prepared according the method of Krieg et al. (1983). After digestion with the appropriate restriction enzymes. 10 μg per sample was electrophoresed in 0.8% (w/v) agarose gels, transferred to Hybond-N$^+$ membranes (Amersham) and fixed with 0.4 M sodium hydroxide for 10–20 min.

### *Preparation of RNA and Northern blotting*

Total cellular RNA was extracted using a single-step acid phenol protocol (Chomczynski and Sacchi, 1987) and 20 μg per sample electrophoresed in 0.9% (w/v) agarose gels in the presence of 0.66 M formaldehyde. The gels were washed in 50 mM sodium hydroxide in 1 × SSC (0.15 M sodium chloride) for 15 min followed by 10 × SCC for 45 min before transfer to Hybond-N membranes (Amersham) and the RNA fixed by baking for 2 h at 80°C. RNA integrity and equal loading was confirmed by ethidium bromide staining.

### *Plasmids*

The plasmids used for probes were pProSp53 (Matlashewski et al., 1987) which contains a normal human p53 cDNA [amplified by polymerase chain reaction (PCR)], pCM41 containing third exon sequences of human c-*myc* (1.4 kb fragment excised with *ClaI*/*EcoRI*), pLRbRNL (Su Huang et al., 1988) containing a normal human RB1 cDNA (0.9 kb fragment excised with *EcoRI*/*BglII*) and p465.20 (ATCC) containing a normal mouse *jun*B cDNA (excised with *EcoRI*). DNA fragments to be used as probes were gel purified before oligolabelling with [$^{32}$P]dCTP (Prime-It II, Stratagene).

### *Hybridisation conditions*

Both Southern and Northern blots were prehybridised overnight in 5 × SSC, 5 × Denhardt's, 50 μg ml$^{-1}$ sheared, denatured salmon sperm DNA and 0.5% (w/v) sodium dodecyl sulphate (SDS) at 65°C. The blots were probed with $^{32}$P-labelled cDNAs for c-*myc*, *RB1*, *jun*B or p53 in prehybridisation solution for 24 h at 65°C. Blots were washed twice in 2 × SSC, 0.1% (w/v) SDS for 15 min at 65°C, 1 × SSC, 0.1% (w/v) SDS for 15 min at 65°C and, finally, in 0.5 × SSC, 0.1% (w/v) SDS for 10 min at 65°C before autoradiography.

### *Western blot analysis*

In order to detect Rb protein, cell lysates were prepared by washing trypsinised cells (× 2) in ice-cold phosphate-buffered saline (PBS) and resuspending 10$^6$ cells in 10 μl of PBS. An aliquot of 100 μl of lysis buffer [25 mM Tris-HCl pH 7.4, 50 mM sodium chloride, 0.5% sodium deoxycholate, 2% NP-40, 0.2% SDS, 50 μg ml$^{-1}$ aprotinin, 50 μM leupeptin, 1 mM phenylmethylsulphonyl fluoride (PMSF)] was added to the cells, the solution was vortexed and then incubated on ice for 15 min with periodic agitation. The cell debris was removed by microcentrifugation at 13 000 *g* for 15 min at 4°C and the proteins separated on a 7.5% (w/v) gel by SDS–polyacrylamide gel electrophoresis (PAGE). Proteins were Western blotted onto Immobilon-P membranes (Millipore), blocked by overnight incubation in 5% (w/v) dried milk in 25 mM Tris-HCl (pH 8.0) and 125 mM sodium chloride (block buffer) at 4°C. Membranes were probed for pRb with anti-human Rb monoclonal antibody (1:1000 dilution in blocking buffer; RB245; PharMingen) and overnight incubation at 4°C. The membranes were washed in blocking buffer (2 × 5 min), Tris-buffered saline (TBS) (2 × 10 min) and blocking buffer (1 × 10 min). The membranes were then incubated with anti-mouse IgG conjugated with horseradish peroxidase (1:1000 dilution in blocking buffer; Sigma) for 1 h at room temperature, washed in blocking buffer (2 × 5 min) and finally washed in TBS containing 0.1% (v/v) Tween 20 (2 × 10 min). Proteins were detected using the enhanced chemiluminescence (ECL) detection system (Amersham) according to the manufacturer's protocol.

For the analysis of c-*myc* and p53 protein, 1 × 10$^6$ cells were washed in growth medium and then PBS, lysed by boiling in 1 × sample buffer and the proteins resolved on 10% (w/v) gels by SDS–PAGE. The proteins were detected as described above except that the monoclonal antibodies Ab3 for c-*myc* and PAb 1801 for p53 (both 1:1000 dilution in blocking buffer; Oncogene Science) were the primary detection antibodies.

### Results

### C-*myc* *gene amplification and expression*

In order to determine whether the c-*myc* locus was amplified in tumour cells with respect to normal keratinocytes, Southern hybridisation of genomic DNA was carried out using a human c-*myc* third exon probe. In contrast to normal keratinocytes, amplification of the c-*myc* gene was observed in all eight of the tumour-derived cell lines (Figure 1).

In order to determine whether amplification of c-*myc* genomic sequences resulted in increased levels of c-*myc* mRNA, Northern blot analysis of total cellular RNA was carried out. As demonstrated in Figure 2, c-*myc* mRNA expression was found to correspond to the level of c-*myc* gene amplification.

The effect of TGF-β1 on c-*myc* transcription in the growth inhibited cell lines H376 and H400 is shown in Figure 3a. Exposure of all responsive cell lines (including normal keratinocytes and H357; data not shown) and two of four partially responsive cell lines (H157, H413) resulted in a rapid (1–5 h) down-regulation of c-*myc* transcription. By contrast, c-*myc* transcription was unaltered in H103 and T-45 (partially responsive to TGF-β1) and H314 (refractory to TGF-β1) cell lines following treatment with exogenous TGF-β1 (2 ng ml$^{-1}$) for up to 24 h (Figure 3b). Examining the effect of TGF-β1 (2 ng ml$^{-1}$) for 24 h on c-*myc* protein exp-

**Figure 1** Southern blot analysis of c-*myc* in tumour-derived human oral keratinocytes showing gene amplification in all cell lines compared with normal DNA. Genomic DNA from normal and tumour-derived keratinocytes (10 μg) and from the human promyeocytic leukaemia cell line HL60 (5 μg used as a positive control) was digested to completion with *EcoRI* before electrophoresis.

ression, using Western blot analysis, confirmed the results obtained by Northern blotting. This is shown in Figure 3c for H357, H376 (responsive), H103 (partially inhibited) and H314 (refractory).

*Analysis of Rb*

Before establishing the effects of TGF-β1 on pRb expression and phosphorylation patterns, it was necessary to determine the presence of the *RB1* gene and ensure its structural integrity. Southern hybridisation of genomic DNA with the *RB1* probe revealed that the *RB1* gene was present in normal keratinocytes and all tumour-derived cell lines. There was no evidence of gross structural abnormalities (not shown).

To examine *RB1* transcription in normal and carcinoma cells, total RNA was Northern blotted and hybridised with the *RB1* cDNA probe. All cell lines expressed *RB1* mRNA irrespective of their TGF-β1 phenotype. No alteration to the levels of *RB1* transcripts was observed when cells were exposed to exogenous TGF-β1 (2 ng $ml^{-1}$ for up to 24 h, data not shown).

As TGF-β1 was found to exert no control on *RB1* transcription, the phosphorylation state of the Rb protein in response to TGF-β1 was examined by Western blot analysis. TGF-β1 (10 ng $ml^{-1}$ for 48 h) caused the accumulation of the underphosphorylated form of the Rb protein in all tumour-derived keratinocytes. This is shown in Figure 4 for the markedly growth-inhibited cell line H400, the partially inhibited cell lines H103, H157 and H413 and the refractory cell line H314.

*Effect of TGF-β1 on junB expression*

Previous studies have indicated that TGF-β can modulate the expression of the *jun*B proto-oncogene in cells which are unresponsive to TGF-β-induced growth inhibition. We therefore examined the effect of TGF-β1 on *jun*B transcription in a growth responsive cell line (H400) and the refractory cell line (H314) in which TGF-β1 did not down-regulate c-*myc* expression. Treatment of the cells with TGF-β1 (2 ng $ml^{-1}$) resulted in a rapid (0–2 h) increase in *jun*B expression (Figure 5).

*Effect of TGF-β1 exposure on cells expressing normal and mutant p53 species*

As previous studies have suggested that cells expressing p53 mutants may exhibit an impaired response to TGF-β1, the effect of TGF-β1 on p53 transcription was investigated by Northern blot analysis. Exogenous TGF-β1 (2 ng $ml^{-1}$ for 24 h) did not affect p53 mRNA expression in normal keratinocytes or in any of the tumour-derived cell lines (data not shown).

There were no alterations in p53 protein levels in tumour-derived cell lines (six of eight cell lines) expressing stable p53 protein following treatment with TGF-β1 using 2 ng $ml^{-1}$ for 24 h and 10 ng $ml^{-1}$ for 48 h (data not shown). Normal keratinocytes, H376 and H413 did not express detectable

**Figure 2** Northern blot analysis of c-*myc* mRNA (**a**), demonstrating that an increase in gene dosage broadly correlates with higher transcription levels. Equal loading was confirmed by ethidium bromide staining (**b**).

**Figure 3** Effect of TGF-β1 on c-*myc* expression. mRNA levels were analysed by Northern blotting in the absence (–) or presence (+) of 2 ng $ml^{-1}$ TGF-β1 for 5 h (**a**) or 24 h (**b**). c-*myc* transcription was down-regulated in all cell lines except H103, T-45 (partially inhibited) and H314 (refractory) in response to TGF-β1. Equal loading was confirmed by ethidium bromide staining (not shown). Similar results were obtained by Western blot analysis (**c**). Protein levels were determined in lysates of $1 \times 10^6$ cells treated with 2 ng $ml^{-1}$ TGF-β1 for 24 h.

**Figure 4** The effect of TGF-β1 on the phosphorylation state of the Rb protein, as determined by Western blot analysis. Lysates were prepared from $1 \times 10^6$ cells in the absence (–) or presence (+) of 10 ng $ml^{-1}$ TGF-β1 for 48 h. An accumulation of underphosphorylated pRb was observed in all cell lines in response to TGF-β1.

**Figure 5** Time course of *jun*B transcription in response to exogenous TGF-β1. mRNA was analysed by Northern blotting in the absence (–) or presence (+) of 2 ng $ml^{-1}$ TGF-β1 for the indicated times (h). TGF-β1 enhanced *jun*B transcription in both H400 (growth inhibited) and H314 (refractory).

levels of p53 (Yeudall et al., 1995) and, therefore, TGF-β1-mediated effects could not be detected.

In addition, the HaCaT keratinocyte cell line (in which down-regulation of p53 protein expression has been demonstrated previously following treatment with TGF-β1; Landesman et al., 1992) was used as a control in these experiments. In the present study, no effect on p53 expression was observed in HaCaT cells in response to TGF-β1 exposure.

### Discussion

The mechanisms by which TGF-β1 mediate growth inhibition after binding to specific cell surface receptors remain unknown. In the present study, we have examined the effect of TGF-β1 on c-*myc*, *RB1*, *jun* B and p53 expression in normal human oral keratinocytes and tumour-derived cell lines which exhibit a range of inhibitory responses to TGF-β1 (Prime et al., 1994). The data are summarised in Table I, together with previously published findings concerning these cell lines.

In normal human keratinocytes, TGF-β1 reversibly inhibits cell growth in the late $G_1$ phase of the cell cycle (Munger et al., 1992) and this is thought to be associated with a marked reduction of c-*myc* expression (Pietenpol et al., 1990*a*). In the present study, all eight tumour-derived cell lines exhibited c-*myc* gene amplification and a corresponding increase in mRNA expression. We have shown previously that six of the eight cell lines in this study contained isochromosome 8q and the others harboured increased copies of chromosome 8, implying an increase in copy number of the c-*myc* gene (8q24). The results of the present study are consistent with previous observations relating c-*myc* gene amplification to overexpression (Alitalo et al., 1987). If c-*myc* levels mediate the growth inhibitory function of TGF-β1, it might be expected that overexpression of c-*myc* protein would enable tumour cells to overcome TGF-β1-induced growth arrest. The present study demonstrates that c-*myc* mRNA overexpression does not abrogate TGF-β1 growth inhibition. Further, we demonstrate that c-*myc* down-regulation by TGF-β1 corresponds to growth inhibition by this ligand in six of eight tumour-derived cell lines. In two cell lines which were partially responsive to TGF-β1 (H103, T-45), however, c-*myc* expression was not down-regulated. These data can be interpreted by suggesting that TGF-β1 can inhibit cell cycle progression outwith c-*myc* regulation.

The fact that HPV 16 E7, the SV40 T antigen and the adenovirus E1a protein not only bind and inactivate the underphosphorylated Rb protein ($pRb^{105}$) but also prevent the down-regulation of c-*myc* expression by TGF-β1, has led to the concept that the Rb protein is involved in the transcriptional regulation of c-*myc* by TGF-β1 (Pietenpol et al., 1990*b*). In the present study, we examined the presence of the *RB1* gene because previous observations indicated a loss of chromosome 13, where the *RB1* gene is located (13q 14.1), in H314 (Patel et al., 1993). The results of this study demonstrate that the *RB1* gene was present in all cell lines, suggesting a chromosome translocation in H314. Whilst *RB1* mRNA was unaltered by TGF-β1, the ligand caused the accumulation of the underphosphorylated form of the Rb protein in all of the tumour-derived cell lines irrespective of both the degree of TGF-β1-induced growth inhibition and TGF-β1-c-*myc* down-regulation. Our data indicate that Rb may not be fundamental to the TGF-β-c-*myc* pathway and, in turn, may function independently of ligand-induced growth inhibition. In H314, for example, the Rb pathway was intact but the c-*myc* pathway was abrogated and yet the cell line continued to cycle in the presence of exogenous TGF-β1. These findings are in accordance with studies showing down-regulation of c-*myc* and growth inhibition following TGF-β treatment in cells that express a non-functional pRb (Zentella et al., 1991; Koike et al., 1994). Whether there is a defect in the signalling pathway between Rb and c-*myc* in H314, such that TGF-β1 alters pRb phosphorylation before c-*myc* transcription control, is currently not known. The cell lines used in the present study all contained mutant p53 (Yeudall et al., 1995) indicating that the cyclin D–cdk4 and cyclin E–cdk2 pathways, which are p53 dependent (Hunter and Pines, 1994; Ewen et al., 1995), are likely to be non-functional. TGF-β1, therefore, is likely to regulate Rb phosphorylation independently of these pathways. The question of how TGF-β regulates c-*myc* down-regulation independently of Rb phosphorylation and how TGF-β-induced growth control can occur independently of c-*myc* regulation remains an enigma. One possible mechanism is the regulation of the cell cycle via the cyclin-inhibitory protein p21, which binds and inactivates components of the DNA replicatory machinery (Waga et al., 1994); interestingly, recent data indicate that p21 acts independently of p53 (Parker et al., 1995).

The results of the present study indicate that control of *jun*B expression is also independent of cellular proliferation, confirming previous observations in mink lung epithelial cells (Chen et al., 1993). TGF-β is known to bind to three high-affinity cell surface receptors termed types I, II and III (Massague, 1992). It has previously been demonstrated that both type I and II receptors are required for TGF-β signalling (Laiho et al., 1990b; Wrana et al., 1992). Extending these findings, Chen et al. (1993) have proposed that there are different TGF-β1 signalling pathways mediated by type I and II receptors, the former involving the induction of *jun*B expression and the elaboration of extracellular matrices and the latter including c-*myc* down-regulation and inhibition of cell growth. It is tempting to speculate that both *jun*B and Rb are involved in signalling pathways leading to extracellular matrix (ECM) elaboration, but this obviously requires experimental confirmation. All the cell lines in the present study express both type I and II receptors in variable proportions (Prime et al., 1994). Our results indicate that the expression of one specific TGF-β receptor does not correlate with

**Table I** Characteristics of tumour-derived human oral keratinocytes

| *Cell line* | *Differentiation*[a] | *Tumorigenicity*[b] | *TGF-β1*[c] *response* (Prime et al., 1994) | TGF-*β1*-*mediated* c-myc *down-regulation* | *TGF-β1-mediated accumulation of underphosphorylated pRB* | *Ha*-Ras *mutation (codon)* (Yeudall et al., 1993) | p53 mutation codon (exon) (Yeudall et al., 1995) |
|---|---|---|---|---|---|---|---|
| Normal | – | NT | + + + | + | + | – | – |
| 103 | M | T | + | – | + | – | 244 (7) G–T |
| 157 | M | NT | + | + | + | – | 306 (8) G–A |
| 314 | P | T | – | – | + | – | 176 (5) G–T, 373 (11) A–G |
| 357 | W | T | + + + | + | + | 13 G–A, 61 A–G | 110 (4) G–A |
| 376 | M | NT | + + + | + | + | – | 266 (8) G–T |
| 400 | M | NT | + + + | + | + | – | 283 (8) C–G |
| 413 | W | NT | + | + | + | – | 68 (4) A–G |
| T-45 | ND | T | + | – | + | – | 110 (4) G–T |

[a]Keratin profiles of differentiation: W, well-differentiated; M, moderately differentiated; P, poorly differentiated; ND, not done. [b]Approximately $1 \times 10^7$ cells were transplanted subcutaneously into 4- to 6-week-old male athymic (nu/nu; Balb/C) mice. Animals were killed following tumour formation or after 6 months. T, tumorigenic; NT, non-tumorigenic. [c]Cellular response to exogenous TGF-β1 as determined by [$^3$H]thymidine incorporation assays. + + +, Markedly inhibited; +, partially inhibited; –, refractory.

the control of c-*myc*, Rb or *jun*B but do not exclude the possibility of interdependent receptors with separate signalling pathways. The possibility of divergent receptor-mediated TGF-β signal transduction mechanisms, however, is currently being questioned (Weiser *et al.*, 1994; Wrana *et al.*, 1994) and is likely to be an area of intense study in the future.

We have demonstrated previously that all of the cell lines in the present study contain mutant p53 in either a missense (H103, H314, H357, H400, H413, T-45) or nonsense (H157, H376) form. The fact that the nonsense mutations in H157 and H376 resulted in a truncated protein (Yeudall *et al.*, 1995) and the cells are markedly inhibited by TGF-β1 (Prime *et al.*, 1994) indicates that p53-independent pathways are likely to be involved in TGF-β signal transduction. This conclusion differs from previous observations (Gerwin *et al.*, 1992; Landesman *et al.*, 1992) and, indeed, Landesman *et al.* (1992) demonstrated a reduction in p53 protein levels in the immortalised human HaCaT keratinocyte cell line following TGF-β1 treatment. In the present study, TGF-β1 did not alter p53 protein levels in either the tumour-derived keratinocyte cell lines or the HaCaT cell line. Whether the discrepancies between the results of the present study and those of Landesman *et al.* (1992) reflect culture conditions and/or the use of different monoclonal antibodies (present study, PAb 1801; Landesman *et al.*, 1992 PAb 421), both of which detect mutant and wild-type p53, is currently not known.

The relationship between activation of *ras* genes and the cellular responsiveness to TGF-β is unclear. We have shown previously that transfection of the mutant cellular Ha-*ras* gene into the human immortalised HaCaT keratinocyte line resulted in a progressive loss of response to TGF-β1 (Game *et al.*, 1992), findings that are entirely consistent with p21-*ras* being involved in TGF-β1 signal transduction (Howe *et al.*, 1993). The results of the present study indicate that TGF-β1 can inhibit epithelial cell growth despite the presence of Ha-*ras* mutations (H357); previous studies have shown the presence of viral and cellular mutant *ras* gene in TGF-β-sensitive cell lines (Manning *et al.*, 1991; Missero *et al.*, 1991). The data of the present study indicate that TGF-β1 signal transduction can occur independently of *ras* mutation and support the findings of Yan *et al.* (1994) showing that TGF-β1 can activate two different signal transduction pathways, one *ras* dependent and another *ras* independent.

Elucidating the mechansims that control the cell cycle is fundamental to an understanding of cell behaviour and malignancy. The results of the present study indicate that TGF-β-induced growth control can exist independently of the presence of mutant p53 and the control of Rb phosphorylation and c-*myc* down-regulation in tumour-derived human oral keratinocytes. Taken together, the results suggest that multiple mechanisms control TGF-β growth inhibition and that the abrogation of one pathway does not necessarily lead to loss of TGF-β-induced growth control.

**Acknowledgements**
The authors wish to thank Mr AD Fathers and Mrs A Stone for their excellent technical assistance and Mrs S Parker for the preparation of the manuscript. This study was supported by an MRC project grant (G9123775 SD) and Denman's Charitable Trust.

### References

ALITALO K, KOSKINEN P, MAKELA TP, SASKELA K, SISTONEN L AND WINQVIST R. (1987). *myc* oncogenes: activation and amplification. *Biochim. Biophys. Acta*, **907**, 1–32.

CHEN RH, EBNER R AND DERYNCK R. (1993). Inactivation of the type II receptor reveals two receptor pathways for the diverse TGF-β activities. *Science*, **260**, 1335–1338.

CHOMCZYNSKI P AND SACCHI N. (1987). Single-step method of RNA isolation by acid guandinium thiocynate phenol-chloroform extraction. *Ann. Biochem.*, **162**, 156–159.

COFFEY RJ, BASCOM CC, SIPES NJ, GRAVES-DEAL R, WEISSMAN BE AND MOSES HL. (1988). Selective inhibition of growth-related gene expression in murine keratinocytes by transforming growth factor β. *Mol. Cell. Biol.*, **8**, 3088–3093.

EWEN ME, OLIVER CJ, SLUSS HK, MILLER SJ AND PEEPER DS. (1995). p53-dependent repression of CDK4 translation in TGF-β-induced $G_1$ cell-cycle arrest. *Genes Dev.*, **9**, 204–217.

FYNAN TM AND REISS M. (1993). Resistance to inhibition of cell growth by transforming growth factor-β and its role in oncogenesis. *Crit. Rev. Oncogenesis*, **4**, 493–540.

GAME SM, HUELSEN A, PATEL V, DONNELLY M, YEUDALL WA, STONE A, FUSENIG NE AND PRIME SS. (1992). Progressive abrogation of TGF-β1 and EGF growth control is associated with tumour progression in *ras*-transfected human keratinocytes. *Int. J. Cancer*, **52**, 461–470.

GERWIN BI, SPILLARE E, FORRESTIER K, LEHMAN TA, KISPERT J, WELSH JA, PFEIFER AMA, LECHNER JF, BAKER SJ, VOGELSTEIN B AND HARRIS CC. (1992). Mutant p53 can induce tumorigenic conversion of human bronchial epithelial cells and reduce their responsiveness to a negative growth factor, transforming growth factor β1. *Proc. Natl Acad. Sci. USA*, **89**, 2759–2763.

HOWER PH, DOBROWOLSKI SF, REDDY KB AND STACEY DW. (1993). Release from $G_1$ growth arrest by transforming growth factor β1 requires cellular *ras* activity. *J. Biol. Chem.*, **269**, 21448–21452.

HUNTER T AND PINES J. (1994). Cyclins and cancer II: Cyclin D and CDK inhibitors come of age. *Cell*, **79**, 573–582.

KIMCHI A, WANG XF, WEINBERG RA, CHEIFETZ S AND MASSAGNE J. (1988). Absence of TGF-Beta receptors and growth inhibitory responses in retinoblastoma cells. *Science*, **240**, 196–199.

KOIKE M, ISHINO K, HUH N AND KUROKI T. (1994). Growth inhibition of SV40-transformed human keratinocytes by TGF-βs is not linked to dephosphorylation of the Rb gene product. *Biochem. Biophys. Res. Commun.*, **201**, 673–681.

KREIG P, AMTMANN E AND SUAER G. (1983). The simultaneous extraction of high molecular weight DNA and of RNA from solid tumors. *Ann. Biochem.*, **134**, 288–294.

LAIHO M, DECAPRIO JA, LUDLOW JW, LIVINGSTON DM AND MASSAGUE J. (1990*a*). Growth inhibition by TGF-β linked to suppression of retinoblastoma protein phosphorylation. *Cell*, **62**, 175–185.

LAIHO M, WEIS FMB AND MASSAGUE J. (1990*b*). Concomitant loss of transforming growth factor (TGF)-β receptors types I and II in TGF-β resistant cell mutants implicates both receptor types in signal transduction. *J. Biol. Chem.*, **265**, 18518–18524.

LAIHO M, WIES FMB, BOYD FT, IGNOTZ RA AND MASSAGNE J. (1991). Responsiveness to transforming growth factor-β (TGF-β) restored by genetic complementation between cells defective in TGF-β receptors I and II. *J. Biol. Chem.*, **266**, 9108–9112.

LANDESMAN Y, PAGANO M, DRAETTA G, FUSENIG NE AND KIMCHI A. (1992). Modifications of cell cycle controlling nuclear proteins by transforming growth factor β in the HaCaT keratinocyte cell line. *Oncogene*, **7**, 1661–1665.

MANNING AM, WILLIAMS AC, GAME SM AND PARASKEVA C. (1991). Differential sensitivity of human colonic adenoma and carcinoma cells to transforming growth factor beta (TGF-beta): Conversion of an adenoma cell line to a tumorigenic phenotype is accompanied by a reduced response to the inhibitory effects of TGF-beta. *Oncogene*, **6**, 1471–1476.

MASSAGUE J. (1992). Receptors for the TGF-β family. *Cell*, **69**, 1067–1070.

MATLASHEWSKI GJ, TUCK S, PIM D, LAMB P, SCHNEIDER J AND CRAWFORD LV. (1987). Primary structure polymorphism at amino-acid residue-72 of human p53. *Mol. Cell. Biol.*, **7**, 961–963.

MISSERO C, CAJAL SRY AND DOTTO GP. (1991). Escape from transforming growth factor β control and oncogene cooperation in skin tumour development. *Proc. Natl Acad. Sci. USA*, **88**, 9613–9617.

MUNGER K, PIETENPOL JA, PITTELKOW MR, HOLT JT AND MOSES HL. (1992). Transforming growth factor β1 regulation of c-*myc* expression, pRB phosphorylation, and cell cycle progression in keratinocytes. *Cell Growth Differ.*, **3**, 291–298.

PARKER SB, EICHELE G, ZHANG P, RAWLS A, SANDS AT, BRADLEY A, OLSON EN, HARPER JW AND ELLEDGE SJ. (1995). p53-independent expression of $p21^{Cip1}$ in muscle and other terminally differentiating cells. *Science*, **267**, 1024–1027.

PARKINSON EK AND YEUDALL WA. (1991). The culture of primary tumours from human epidermis. In *Primary Cultures for Human tumour Biopsies: a Handbook*. Masters J (ed.), pp. 187–197. Kluwer: Boston.

PATEL V, YEUDALL WA, GARDNER A, MUTLU S, SCULLY C AND PRIME SS. (1993). Consistent chromosomal anomalies in keratinocyte cell lines derived from untreated malignant lesions of the oral cavity. *Genes. Chrom. Cancer*, **7**, 109–115.

PIETENPOL JA, HOLT JT, STEIN RW AND MOSES HL. (1990*a*). Transforming growth factor-β suppression of c-*myc* gene transcription: role in inhibition of keratinocyte proliferation. *Proc. Natl Acad. Sci. USA.*, **87**, 3758–3762.

PIETENPOL JA, STEIN RW, MORAN P, YACIUK R, SCHLEGEL R, LYONS RM, PITTELKOW MR, MUNGER K, HOWLEY PM AND MOSES HL. (1990*b*). TGF-β1 inhibition of c-*myc* transcription and growth in keratinocytes is abrogated by viral transforming proteins with pRB binding domains. *Cell*, **61**, 777–785.

PRIME SS, HIXON SVR, CRANE IJ, STONE A, MATTHEWS JB, MAITLAND NJ, REMNANT L, POWELL SK, GAME SM AND SCULLY C. (1990). The behaviour of human oral squamous cell carcinoma in cell culture. *J. Pathol.*, **160**, 259–269.

PRIME SS, MATTHEWS JB, PATEL V, GAME SM, DONNELLY M, STONE AS, PATERSON IC, SANDY JR AND YEUDALL WA. (1994). TGF-β receptor regulation mediates the response to exogenous ligand but is independent of the degree of cellular differentiation in human oral keratinocytes. *Int. J. Cancer*, **56**, 406–412.

SEGARINI PR, ROSEN DM AND SEYEDIN SM. (1989). Binding of TGF-β to cell surface proteins varies with cell type. *Mol. Endocrinol.*, **3**, 261–272.

SU HUANG HJ, YEE JK, SHEW JY, CHEN PL, BROOKSTEIN R, FRIEDMAN T, LEE EYHP AND LEE WH. (1988). Suppression of the neoplastic phenotype by replacement of the RB gene in human cancer cells. *Science*, **242**, 1563–1566.

WAGA S, HANNON GJ, BEACH D AND STILLMAN B. (1994). The p21 cyclin-dependent kinase inhibitor directly controls DNA replication with PCNA. *Nature*, **369**, 574–578.

WEISER R, ATTISANO L, WRANA JL AND MASSAGUE J. (1993). Signaling activity of transforming growth factor β type II receptors lacking specific domains in the cytoplasmic region. *Mol. Cell. Biol.*, **13**, 7239–7247.

WRANA JL, ATTISANO L, CARCAMO J, ZENTELLA A, DOODY J, LAIHO M, WANG X-F AND MASSAGUE J. (1992). TGF-β signals through a heteromeric protein kinase receptor complex. *Cell*, **71**, 1003–1014.

WRANA JL, ATTISANO L, WEISER R, VENTURA F AND MASSAGUE J. (1994). Mechanism of activation of the TGF-β receptor. *Nature*, **370**, 341–347.

YAN Z, WINAWER S AND FRIEDMAN E. (1994). Two different signal transduction pathways can be activated by transforming growth factor-β1 in epithelial cells. *J. Biol. Chem.*, **269**, 13231–13237.

YEUDALL WA, TORRANCE LK, ELSEGOOD KA, SPEIGHT P, SCULLY C AND PRIME SS. (1993). *ras* gene point mutation is a rare event in premalignant and malignant lesions of the oral cavity. *Oral Oncol. Eur. J. Cancer*, **28B**, 63–68.

YEUDALL WA, PATERSON IC, PATEL V AND PRIME SS. (1995). Presence of human papillomavirus sequences in tumour-derived human oral keratinocytes expressing mutant p53 protein. *Oral Oncol. Eur J. Cancer*, **31B**, 136–143.

ZENTELLA A, WEIS, FMB, RALPH DA, LAIHO M AND MASSAGUE J. (1992). Early gene responses to transforming growth factor-β in cells lacking growth-suppressive RB function. *Mol. Cell. Biol.*, **11**, 4952–4958.